# Genomic Characterization of Extremely Antibiotic-Resistant Strains of *Pseudomonas aeruginosa* Isolated from Patients of a Clinic in Sincelejo, Colombia

**DOI:** 10.3390/biotech14010021

**Published:** 2025-03-16

**Authors:** Nerlis Pajaro-Castro, Erick Diaz-Morales, Kenia Hoyos

**Affiliations:** 1 Medical and Pharmaceutical Sciences Group, Faculty of Health Sciences, University of Sucre, Sincelejo 700001, Sucre, Colombia; 2 Salud Social Clinic, Sincelejo 700001, Sucre, Colombia; erick76dm@yahoo.es (E.D.-M.); kmhoyosgonzalez@gmail.com (K.H.)

**Keywords:** sequencing, genome, resistance, clinical strains, *P. aeruginosa*, multidrug-resistant

## Abstract

*Pseudomonas aeruginosa* is an opportunistic pathogen classified as a priority and a great public health concern; therefore, this research focuses on the genomic characterization of extremely resistant strains of *P. aeruginosa* isolated from patients in a clinic in Sincelejo, Colombia. Seven strains were analyzed by whole genome sequencing using the Illumina NovaSeq platform, with a focus on the identification of resistance genes and virulence factors through the CARD and VFDB databases. An ANI (Average Nucleotide Identity) analysis was carried out to determine the genetic relationship between the strains, complemented by a phylogenomic analysis to place the strains in different evolutionary clades. The results revealed that six of the strains are of Colombian origin, while one strain (547256) belongs to the high-risk clone ST773, previously unidentified in Colombia. Genome size ranged from 6 to 7.4 Mbp, indicating differences in genetic content among strains. Phylogenomic analysis confirmed that five strains belong to a multidrug-resistant (MDR) group, while one strain (572897) showed high alignment with a laboratory strain, and strain 547256 was not associated with any specific clade. Clinically, 100% of strains showed carbapenem resistance, resistance genes, and virulence factors that make them difficult to treat. This study provides key insights into the genetic diversity and resistance patterns of *P. aeruginosa* in this region, underscoring the need to monitor high-risk clones and optimize therapeutic strategies.

## 1. Introduction

*Pseudomonas aeruginosa* is an opportunistic, gram-negative bacterium associated with serious infections in humans due to its pathogenicity and ability to develop resistance to almost all drugs [1]. This microorganism is responsible for high morbidity and mortality rates worldwide and is involved in a wide variety of infections, such as urinary tract infections, pneumonia, surgical wound infections, endocarditis and septicemia, being especially frequent in intensive care units [2,3]. It is highly prevalent in hospital environments, particularly in areas exposed to daily disinfectants and units that receive patients with diverse antimicrobial resistance profiles. Its ability to persist on inanimate surfaces and medical devices is due to its remarkable resistance to biocides, biofilm formation, and genetic plasticity, which facilitates the acquisition of resistance determinants. Additionally, the selective pressure exerted by the constant use of antimicrobials and disinfectants promotes the survival of multidrug-resistant strains, making it a significant challenge for nosocomial infection control [2,3].

In 2017, the World Health Organization (WHO) included carbapenem-resistant *P. aeruginosa* (CR-PA) within the critical group of bacteria that urgently requires research and development of new antibiotics [4]. The global spread of multidrug-resistant (MDR) or extremely drug-resistant (XDR) *P. aeruginosa* has become a threat to public health [5]. In recent years, the increasing prevalence of strains has been observed with rates between 15% and 30% in some geographic areas [5]. The overall prevalence of MDR *P. aeruginosa* in Latin America is higher than 40% and reached 28.4% in Europe [6]. The emergence of carbapenem-resistant isolates constitutes a therapeutic challenge, the problem has worsened in recent years due to the excessive use of antibiotics, which has accelerated the development of survival adaptations in bacteria [7].

The development of resistance mechanisms in *P. aeruginosa* has a significant clinical impact, as it reduces the efficacy of almost all drugs available for its treatment. This leads to high healthcare costs for infected patients, as well as increased mortality and length of hospital stays [8]. Furthermore, patients with MDR *P. aeruginosa* infections have a considerably higher 30-day mortality rate than those infected with non-resistant strains [9]. Several mechanisms are involved in drug resistance, such as the presence of an overexpressed efflux pump, the acquisition of resistance genes through plasmids and transposons, or through mutations in genes encoding porins, efflux pumps, penicillin-binding proteins and inducing the expression of endogenous beta-lactamases such as AmpC, all contributing to resistance to b-lactams, carbapenems, aminoglycosides and fluoroquinolones [10,11].

In a study conducted by [12], they reported that around 47.5% of *P. aeruginosa* isolates were MDR. The resistance rate showed that Imipenem was significantly higher in resistance among clinical isolates (77.8%), then meropenem (61%), aztreonam (42%) and ceftazidime (36%). In Colombia, Ref. [13] reported the presence of the blaKPC-3 gene in clinical isolates of *P. aeruginosa* and the presence of 14 sequence types was detected, with ST111 being the most frequent. Another study reported virulence genes, finding the pilA gene in 48.1% of the isolates and the algD gene in 100% [14].

With a genome of 6.3 million base pairs (Mbp) encoding 5567 genes, *P. aeruginosa* has a remarkably large genome compared to other bacteria [1,15]. Recently, outbreaks of antibiotic-resistant strains in hospital settings have increased, generating greater interest in this topic [16,17]. Current research has provided additional evidence that a small group of MDR and XDR clones are spreading in hospitals worldwide, the most prevalent being ST235, ST111, ST233, ST244, ST357, ST308, ST175, ST277, ST654 and ST298, which have been classified as high-risk clones [18]. In addition, the genome encodes a variety of virulence genes whose expression after infection is associated with disease severity and poor treatment outcomes. These genes include nan, oprL, lasB, toxA, and exoS [19]. The genomic study of bacterial pathogens may be key to designing strategies to combat microbial pathogenesis.

The investigation of virulence factors at the molecular level, in relation to antimicrobial resistance patterns, could open new opportunities or discover innovative approaches for the prevention and treatment of infections, in order to direct infection control interventions and decrease the spread of multi-resistant microorganisms. For monitoring resistance mechanisms in *P. aeruginosa*, several genes can be used as key biomarkers. Among them, genes encoding extended-spectrum β-lactamases (*blaVIM*, *blaIMP*, and *blaNDM*), efflux pumps (*mexA*, *mexB*, *oprM*), and mutations in genes regulating outer membrane permeability (*oprD*) are crucial for tracking resistance evolution. The detection and surveillance of these genes through molecular tools enable the assessment of resistant strain dissemination and the design of effective control strategies in clinical settings [10,11]. Therefore, this research focuses on the genomic characterization of extremely resistant strains of *P. aeruginosa* isolated from patients in a clinic in Sincelejo, Colombia, with the aim of understanding antibiotic resistance profiles and associated virulence mechanisms. The results will fill an important gap related to the genomic sequences of this microorganism in the Sucre region. These characteristics will provide new insights into the epidemiology and genomic mechanisms of this pathogen, which may allow the analysis of resistance trends and the impact on antimicrobial surveillance.

## 2. Materials and Methods

### 2.1. Study Design

We conducted a retrospective experimental study focused on the genomic characterization of XDR strains of *P. aeruginosa* obtained from the Salud Social Clinic in Sincelejo, Sucre, Colombia. As part of routine diagnosis, clinical samples from patients were processed during the period between January 2022 and March 2024. Strains of interest to public health were cryopreserved in skim milk at −20 °C because they were multidrug resistant.

### 2.2. Collection and Processing of Samples

For the selection of *P. aeruginosa* strains, the antimicrobial resistance profiles of the isolates were reviewed during January 2022 and March 2024 in the Whonet software version 5.6. Subsequently, the clinical histories and the results of the reported microbiological tests were reviewed. Seven multidrug-resistant strains were randomly chosen to ensure the representativeness of the isolates in the local clinical context.

### 2.3. Phenotypic Tests

The strains were subjected to a thawing process for 30 min, then sterile calibrated loops were used to inoculate the microorganisms in enriched media (chocolate agar, MDM Cientifica S.A.S., Medellin, Colombia) and differential media (MacConkey agar, MDM Cientifica S.A.S., Medellin, Colombia) by depletion sowing in order to verify the purity and viability of the strains. The cultures were incubated aerobically at 37 °C for 24 h and Gram staining was used for microscopic analysis [20].

The minimum inhibitory concentration (MIC) method was used to evaluate the antibiotic susceptibility of clinical strains. These were processed by adjusting the turbidity of the inocula to McFarland scales between 0.5 and 0.64 using the DensiCHECK instrument (BioMérieux Inc., Durham, NC, USA). AST-93 and AST-403 cards were then used in the VITEK^®^ 2 (BioMérieux Inc., Durham, NC, USA) equipment to assess the antimicrobial susceptibility of the selected strains. Antibiotics used in this study were as follows: beta-lactams/beta-lactamase inhibitors (piperacillin/tazobactam, ceftazidime/avibactam), cephalosporins (cefazolin, ceftazidime, cefepime), monobactams (aztreonam), carbapenems (meropenem), aminoglycosides (amikacin), and fluoroquinolones (ciprofloxacin). The results were analyzed using the breakpoints described in the recommendations of the M100 manual of the Clinical and Laboratory Standards Institute (CLSI) [21]. Taking into account the MIC in μg/mL obtained for each antibiotic, the results were classified as susceptible (S), intermediate (I), and resistant (R). The strains evaluated were classified as multidrug-resistant (MDR) for those that were shown to be resistant to an antibiotic from ≥ three classes, and as extensively drug-resistant (XDR) for those that were shown to be resistant to an antibiotic from ≥ six classes studied. Qualitative detection of carbapenemase-producing bacteria was performed using the Rapidec Carba NP test (BioMerieux, Marcy L’Etoile France), which is a colorimetric test consisting of the direct detection of carbapenem hydrolysis. The cards were controlled using the ATCC *P. aeruginosa* 27853 control strain.

### 2.4. Genotypic Testing

Genomic DNA isolation and whole genome sequencing:

The isolates were sent to the National Center for Genomic Sequencing (CNSG) of the University of Antioquia for whole genome sequencing. Genomic DNA (gDNA) extraction was performed using the Qiagen DNeasy PowerLyzer PowerSoil Kit (QIAGEN GmbH, Hilde, Germany), following the kit manufacturer’s recommendations. The extracted gDNA was quantified by light absorption at 260 nm using the NanoDrop™ 2000 (Thermo Scientific™, Wilmington, DE, USA) and frozen at −20 °C for subsequent genomic sequencing experiments. Genomic sequencing of the gDNA extracted from the bacterial strain was carried out using Truseq Nano DNA libraries (350) and the Illumina NovaSeq platform, through which 150-base paired reads were generated.

### 2.5. Analysis of the Genomic Sequence

The quality analysis of the genomic sequences was performed by the CNSG. In this process, parameters such as base quality, GC content and the presence of adapters were evaluated. The genomic assembly was evaluated considering key metrics, such as genome size, number of contigs, N50 length and percentage of coverage.

Taxonomic assignment was carried out by calculating Average Nucleotide Identity (ANI) using FastANI, comparing the sequences with reference genomes in NCBI RefSeq. Phylogenetic analysis was performed by the CNSG using an in-house script developed by the center, which allows the concatenation of conserved genes and the construction of phylogenetic trees. This analysis facilitated the precise placement of the strains within the phylogenetic groups of *P. aeruginosa*, providing a clear view of their evolutionary relationship with other known strains.

### 2.6. Analysis of Resistance Genes and Virulence Factors

For the identification of antimicrobial resistance genes, the RGI (Resistance Gene Identifier) algorithm based on the Comprehensive Antibiotic Resistance Database (CARD) was used. Simultaneously, the identification of virulence factors was carried out using the Virulence Factor Database (VFDB). Additionally, PlasmidFinder tools were used to identify plasmid sequences in the assembled contigs, in order to evaluate the presence of mobile genetic elements involved in the horizontal transfer of resistance and virulence genes. These analyses provided a comprehensive overview of the genetic mechanisms involved in antimicrobial resistance and pathogenicity in the strains studied.

### 2.7. Ethical Implications

This research is governed by Resolution 8430 of 4 October 1993 and Resolution 2378 of 2008 of the Ministry of Social Protection of Colombia and the Declaration of Helsinki, which declares that this research was retrospective and was carried out with information and cell cultures provided by the Salud Social Clinic. This research is classified as risk free, because retrospective documentary research techniques and methods are used and no intentional intervention or modification of the biological, physiological, psychological or social variables of the individuals participating in the study was performed.

## 3. Results

### 3.1. Phenotypic Characterization

In this study, a search was carried out in the databases of the Salud Social Clinic from 2022 to 2024 of patients with reports of *Pseudomonas aeruginosa*, of which a total of 251 were identified as follows: 91 strains of *P. aeruginosa* in 2022, of which 19.7% are MDR and XDR; in 2023, 103 strains were identified, of which 9.7% were MDR and XDR; and finally in 2024, 21 strains were identified, of which 19% were MDR and XDR. Of these strains, a total of 22 were MDR and XDR according to phenotypic evaluation. The result of the Gram stain showed gram-negative bacilli.

Based on the review of the clinical records of the seven selected samples, it was determined that 14% of the strains came from female patients, and the rest (86%) were from male patients. Most of the patients (86%) were over 50 years of age, and 14% were between 41 and 50 years of age. Regarding the previous use of antibiotic therapy, 51% of the patients had not received antibiotic treatment before the isolation of the strain, while 49% had received it. Of these, 75% were treated with beta-lactam antibiotics and 25% with carbapenems. Regarding hospital stay, 57.1% of the patients had a hospital stay of more than two weeks. One hundred percent of the patients had a permanent intravenous catheter, and 28.6% also had a urinary catheter. Eighty-six percent of cases had a positive urine culture, while 14% had both a positive urine and blood culture. Regarding clinical outcomes, 86% of patients were discharged, and 14% died. All strains (100%) showed resistance to carbapenems. Regarding the timing of infections, 71.4% of patients had a positive urine culture at admission, and 28.6% had a positive urine culture after hospital admission. The indicated treatment varied among patients: 42.9% received meropenem (2 g intravenously every 8 h, in a 3 h infusion) in combination with colistin (150 mg intravenously every 12 h for 10 days), while the remaining 57.1% were treated with ceftazidime-avibactam (2.5 g intravenously every 8 h, in a 3 h infusion) and aztreonam (2 g intravenously every 8 h) for 10 days. Geographically, 100% of the patients were from municipalities outside of Sincelejo, with one from the department of Bolívar and the rest from the department of Sucre.

The results of antimicrobial susceptibility testing using Vitek cards are shown in Table 1. All seven bacterial strains tested were shown to be extensively drug-resistant (XDR) because they were resistant to one antibiotic from ≥ six of the tested classes. For all strains, the phenotype high-level cephalosporinase + carbapenemase resistant (imper), carbapenemase, ESBL (extended spectrum beta-lactamases) + carbapenemase resistant (imper) were identified. The identified carbapenem-resistant *P. aeruginosa* (CRPA) strains were shown to be resistant to the tested antimicrobials.

### 3.2. Genotypic Characterization

The gDNA was sequenced using the Illumina NovaSeq platform, obtaining the results shown in Table 2. Between 19,829,196 and 46,881,312 paired-end reads of 150 base pairs were generated, with a high-quality read rate (Q30) in a range of 92.44 to 94.53% and an average coverage depth between 207,998 and 484,218X. By analyzing Table 2, it can be seen that all strains belong to the *P. aeruginosa* species. The content of Guanine-Cytosine (GC) in all strains studied was similar, ranging between 65.6% and 66.0%. In addition, a high degree of genomic completeness was evident in all samples, with values between 97.94% and 99.86%. This indicates that the genome sequencing and assembly process was successful and produced an almost complete genome of the clinical samples of the strains. The number of protein-coding sequences (CDS) is similar in all strains, with values between 6546 and 6838. The total number of sequences varies between 134 (strain 547256) and 233 (strain 572897). The total genome length varies between 6,982,541 (strain 637345) and 7,073,876 (strain 572897). The N50 size varies between 205,279 (strain 637345) and 284,837 (strain 629590). Differences in the total number of sequences and total genome length (6,982,541 to 7,109,622 bp) could indicate the presence of insertions, deletions or genomic rearrangements between strains. These changes in genome structure may be the result of mutational events or acquisition of mobile genetic elements. Differences in the number of CDSs (6546 to 6838) could indicate the presence of accessory genes or gene loss in some strains. These additional or missing genes may confer specific phenotypic characteristics to each strain, such as the ability to utilize different carbon sources, stress resistance or virulence. The genome contains 70 (544871, 572879 and 6373450), 68 (547256) and 67 (629590, 6373450 and 645441) transfer RNA genes, three (544871, 572879, 629590, 6373450, 645441 and 6373450), four (547256) ribosomal RNA genes and two CRISPR arrays (547256).

Strain 34Pae36 exhibits high nucleotide similarity with the *P. aeruginosa* strains studied according to the analysis performed (Table 3). The number of single nucleotide polymorphisms (SNPs) varies considerably between strains, from ten to thousands of SNPs. These differences in the number of SNPs reflect the degree of genetic divergence between strains, which may be due to mutational events, acquisition of mobile genetic elements or adaptation to different environments. In the comparative analysis of the seven *P. aeruginosa* strains, six of the strains aligned with the reference strain 34Pae36 in more than 97% of their genomic sequences, and one of the strains (547256) aligned with the reference strain ST773 in 91.8%. Strain 34Pae36 belongs to a clinical *P. aeruginosa* from Bogotá, Colombia [22]. Strain 547256 belongs to the clonal lineage ST773, which has been reported in several countries and is associated with severe infections and resistance to multiple antibiotics. Previous studies have identified ST773 as a high-risk clone of *P. aeruginosa*, with an increased ability to cause infections and increased antibiotic resistance compared to other lineages. Strain ST773 has been associated with nosocomial outbreaks and has been found in respiratory tract infections, wounds, and blood samples, indicating its potential to cause invasive infections [23].

**Figure 1:**
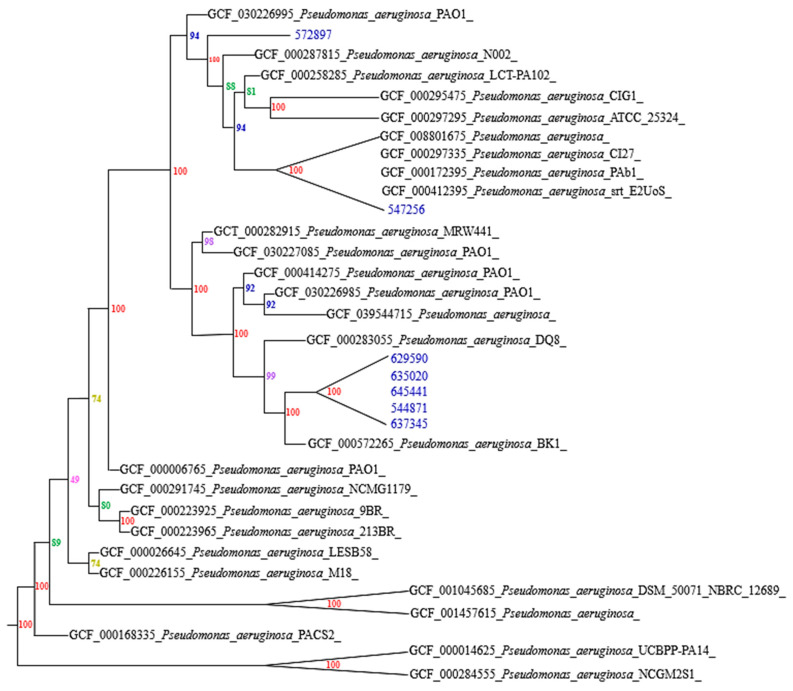
Phylogenetic analysis of the strains studied.

Phylogenomic analysis based on 113 concatenated genes confirmed the classification of the strains within the *P. aeruginosa* clade (Figure 1), with high support values on the branches, reinforcing the robustness of the observed phylogenetic clustering. Phylogenetically closer strains showed lower evolutionary distances and fewer single nucleotide polymorphisms (SNPs), suggesting a recent common origin and/or lower selective pressure in their evolution. This finding is consistent with the hypothesis that strains with phylogenetic proximity share similar phenotypic and genotypic characteristics, possibly reflecting adaptations to specific clinical settings. More phylogenetically distant strains could represent independent introductions into the hospital environment. Phylogenetic analysis showed that strains 629590, 635020, 645441, 544871 and 637345 cluster in the same clade, suggesting significant genetic proximity and possibly a recent common origin related to geographical adaptations. These strains are genetically close to PAO1 BK1, a *P. aeruginosa* MDR strain [24], while strains 572897 and 547256 were located in different clades, indicating that these strains have higher genetic diversity and potentially different evolutionary trajectories. Among them, strain 572897 showed higher genetic closeness to PAO1, a *P. aeruginosa* strain from a laboratory culture [25]. While 547256 did not show genetic closeness to any of the clades, ANI analysis showed its similarity to ST773. These results underline the importance of continued phylogenetic monitoring to understand the evolutionary and dispersion dynamics of *P. aeruginosa* in clinical settings, which is essential for the development of effective control and treatment strategies.

Additionally, a comparative analysis was performed between the different strains of *P. aeruginosa* (Table 4). Most of the comparisons between strains show a high average identity percentage (AvgIdentity) ranging between 96% and 99%. This indicates that the strains are genetically similar, suggesting that they may have similar phenotypic and pathogenic characteristics. The aligned bases are consistently high (around 99,000 in the closest pairs), indicating that extensive sequences have been aligned and that the quality of the alignment is good; however, strain 547256 presents a significantly lower average identity (around 88%) compared to the other strains, indicating that it could be a different lineage or have genetic adaptations that distinguish it from the other more homogeneous strains. There is significant variability in the number of SNPs between strains. For example, the strain pair 547256 and 635020 shows 64,848 SNPs, indicating considerable genetic divergence, in contrast to other pairs that have only a few SNPs. This suggests that 547256 is genetically more distant from the other strains analyzed. We therefore performed a single gene analysis, which revealed that only strains 637345 and 547256 harbored these genes. Among these, nine were identified as virulence-related (pldA/tle5a, exoU, rhsP2, pvdIV, pvdJ, cupC1, pilA, spcU and phzG2), seven were associated with resistance (FosA, arr-2, PDC-385, dfrA22, mphE, APH(3′)-VI and msrE) and two were unknown genes (PA4541 and PLES_RS09940). Finally, the association between the identified resistance genes and the resistance patterns observed in the strains was confirmed (Table 1). Furthermore, resistance genes responsible for conferring resistance to other classes of antimicrobials and antiseptics were detected (Table 5), underlining the wide adaptability of these strains.

It is important to highlight that strain 547256, which is located in a different clade, was found to contain highly important virulence and resistance genes, such as exoU, rhsP2, FosA and pvdJ, which confer a high capacity to cause severe infections and resistance to current antibiotic treatments. This suggests that this strain has developed a set of genetic mechanisms that allow it not only to survive under the selective pressure of antibiotics but also to compete successfully in the clinical environment, which is reflected in its phylogenetic difference from the main group. On the other hand, although strain 637345 belongs to the same clade as the other five strains, it presents unique genes, such as msrE, mphE and APH(3′)-Via, which confer resistance to different antibiotics, highlighting the genetic diversity within the same clade and suggesting that this strain could have acquired these resistance genes through horizontal transfer mechanisms. This genetic variability within a single clade highlights the complexity of the evolution of *P. aeruginosa* in hospital environments, where selective pressure exerted by the use of antibiotics and competition between strains leads to the emergence of lineages with unique resistance and virulence profiles.

On the other hand, antimicrobial resistance and virulence factors were identified in the strains studied, including the genes MexA, MexB, and OprM, which are components of the MexAB-OprM efflux pump system [26]. This confers resistance to multiple classes of antibiotics, including beta-lactams, tetracyclines, chloramphenicol, and fluoroquinolones, which is consistent with what was found phenotypically in the strains studied. AmpC is an inducible beta-lactamase that provides resistance to penicillins and cephalosporins, which was observed in the phenotypic study. BlaOXA, BlaVIM, BlaIMP, are extended-spectrum beta-lactamases (ESBLs) and carbapenemases, which confer resistance to a wide range of beta-lactam antibiotics, including carbapenems [27], which was evidenced phenotypically. Additionally, virulence factors, such as LasR, RhlR, PqsR, which are regulators of quorum signaling, controlled the expression of numerous genes involved in virulence, including those encoding toxins and degradative enzymes. AlgD, AlgU, MucA, and MucB are genes related to alginate production and biofilm formation, crucial features in chronic infections, particularly in patients with cystic fibrosis [28]. ExoS, ExoT, ExoU, and ExoY are type III secretion system (T3SS) inhibitors, which play a key role in evading the host immune system and in cellular cytotoxicity. ToxA, which encodes exotoxin A, is a major virulence factor that inhibits protein synthesis in host cells. PlcH and PlcN are Phospholipases C involved in the degradation of host cell membranes and in the dissemination of infection. PilA, PilB, PilC, and PilD, are components of type IV pili, essential for motility, biofilm formation and adhesion to surfaces [28]. PvdE and PvdS are genes involved in the biosynthesis of siderophores, which are crucial for the acquisition of iron, a limited nutrient during infection. Analysis of clinical strains of *P. aeruginosa* shows a complex and highly adapted resistance and virulence profile. The presence of efflux pumps, such as MexAB-OprM, and the expression of beta-lactamases, such as AmpC and OXA, were identified, demonstrating the common antibiotic resistance mechanisms of these strains [26]. Furthermore, these strains might be uniquely equipped to establish chronic infections, particularly in hospital settings or in patients with pre-existing diseases, such as cystic fibrosis, due to the presence of genes related to biofilm formation and alginate production, such as algD, mucA, mucB and algU. The genes exoS, exoT and exoU were also identified, indicating that these strains encode toxins secreted by the type III secretion system, highlighting their ability to cause significant tissue damage and evade the host immune response, which could explain their success in acute and severe infections. Finally, quorum-sensing genes, such as lasR and rhlR, suggest that these strains can coordinate the expression of virulence factors in response to environmental cues, representing a significant adaptive advantage during infection [28].

## 4. Discussion

*P. aeruginosa* is an ever-present bacterium in the environment. It has been estimated that it colonizes up to 15% of patients in hospitals, and its eradication is difficult, if not impossible. This bacterium primarily affects individuals with weakened immune systems, such as hospitalized patients, those with cystic fibrosis, or individuals undergoing immunosuppressive treatments. Its virulence activity is closely linked to its ability to form biofilms, evade the host immune response, and produce various virulence factors, including elastases, exotoxins, and quorum sensing-regulated molecules. Therefore, efforts should focus on strengthening infection control measures at the hospital level to limit its transmission [29]. Indeed, one study found that the frequency of *P. aeruginosa* was high in patients treated in intensive care unit (ICUs). The results show that resistance levels are excessively high. Because special attention should be paid to monitoring and optimizing the use of antimicrobials to reduce the emergence and spread of pathogens resistant to antimicrobial drugs in ICUs [30], this bacterium is included in the list of priority pathogens by the World Health Organization [31]. In this study, the clinical results presented reflect the challenge posed by extremely resistant *P. aeruginosa* infections, particularly in patients older than 50 years and predominantly male. The high resistance to carbapenems in this study (100%) highlights the severity of these infections in a clinical context, coinciding with previous studies associating the prolonged use of beta-lactam and carbapenem antibiotics with the development of multi-resistant strains [32]. The majority of patients (71.4%) had a positive urine culture upon admission, suggesting a previously acquired infection not exclusively related to hospitalization. This highlights the importance of community or pre-hospital risk factors in the acquisition of extremely resistant *P. aeruginosa* infections. The identification of resistance genes and phylogenetic assignment of these extreme strains underscore the need for continued surveillance and rigorous control of antibiotic use, especially in vulnerable geographic areas, such as municipalities outside Sincelejo.

The ANI analysis revealed that six of the strains evaluated are of Colombian origin and only one (547256) belongs to a high-risk clone ST773 not identified in Colombia so far. In addition, the genome size of the strains varied between 6 and 7.4 Mbp, demonstrating substantial differences in genetic content. While the phylogenomic analysis indicates that five strains belong to MDR strains, one is highly aligned with a laboratory strain (572897) and 547256 is not significantly related to any clade. These differences are due to the fact that they are different methodologies, in which the phylogenomic analysis evaluates evolutionary relationships and can give different results if the strains have significant genomic variations, such as the acquisition of genes by horizontal transfer, which may not be reflected uniformly in all the genes used for the phylogenetic analysis. However, both analyses identify strain 547256 as a unique case. Furthermore, variability in genome size was evidenced, these differences may be related to the acquisition or loss of mobile genetic elements, as observed in cystic fibrosis isolates, where a reduction in genome size was documented due to the loss of non-essential regions for survival in hostile environments [33]. The sequencing process was efficient due to high quality read rates (Q30) ranging from 92.44% to 94.53%, comparable to the results reported by Stover et al. (2000) on the PAO1 reference genome, where high levels of quality and coverage were also evidenced. These parameters are important to ensure accuracy in genome assembly and annotation, which in turn affects the interpretation of genomic data and their application in subsequent studies [13,34].

Brinkman et al., 2021, reported high genome completeness values in their studies on *P. aeruginosa*, which are similar to those observed (97.94% to 99.86%) in the strains analyzed in this study. N50 values ranging from 205,279 to 284,837 bp show good genome assembly [13,35]. The GC content in the analyzed strains, ranging between 65.6% and 66.0%, aligns with the typical content reported for *P. aeruginosa*, which is usually around 66%. This consistency suggests that the strains studied maintain typical genomic features of the species, which is relevant to understand their biology and ecology [36]. Variations in the number of CDSs (6546 to 6838) and in the total genome length (6,982,541 to 7,073,876 bp) may reflect differences in the presence of accessory genes and mobile genetic elements. This is consistent with the findings of [13,37] who pointed out that differences in the genome size of *P. aeruginosa* are largely due to the “accessory genome”, which includes plasmids and other mobile elements These genetic differences could have major implications for the ability of strains to adapt to different environments and selective pressures. The identification of CRISPR systems in the clinical strain 547256 coincides with the findings of [13,38], who also reported the presence of these systems in *P. aeruginosa* strains. CRISPR systems may provide selective advantages in certain environments, offering immunity against phages and plasmids, which could explain their differential appearance among strains and their relevance in the evolution of antibiotic resistance.

Genomic analysis performed on *P. aeruginosa* strains isolated from patients at a clinic in Sincelejo revealed significant diversity in antimicrobial resistance genes and virulence factors, which is consistent with the genomic variability observed in other studies. As in previous research, such as that described by [39], the *P. aeruginosa* genome was found to be highly variable, with the presence of conserved and non-conserved genomic regions, suggesting a high potential for acquiring accessory and unique genes through horizontal gene transfer. In our study, we identified key genes, such as mexA, mexB, mexR, cpxR, and oprM, which are implicated in resistance to multiple classes of antibiotics. These genes, which were also reported in strain EMAR01 [36], are part of the resistance–nodulation–cell division (RND) efflux pump system, reinforcing the ability of these strains to resist a wide range of antimicrobials, a finding that resonates with the observations of [40] on the importance of genomic sequencing to track multi-resistant strains in hospital settings. Furthermore, phylogenomic analysis based on 113 concatenated genes allowed identifying that strains 572897 and 547256, belonging to different clades, possess greater variability in resistance and virulence genes compared to strains of the same clade, suggesting greater genetic diversity among distantly related strains. This finding is in line with the studies by [41], where it was shown that *P. aeruginosa* genotypes can display distinct epidemiological behaviors, even within well-characterized clonal complexes. Finally, the identification of exclusive genetic material in our isolates, which does not present alignment with the genome of the PAO1 strain, highlights the complexity of the *P. aeruginosa* genome. As indicated by [42], although a large part of the genomic content is shared with reference strains, there is always a significant percentage of sequences that are unique to each isolate, which underlines the adaptive capacity of this pathogen and its potential to develop new virulence and resistance characteristics. The results obtained demonstrate the need to continue genomic and phenotypic surveillance of the *P. aeruginosa* bacteria at the hospital level in order to improve treatment strategies and infection control as well as to anticipate the emergence of new XDR strains.

A study by (Zhao et al., 2023), reported high heterogeneity in the genomic structure of strains collected in hospital settings in China. In this study, high genetic variability was observed in *P. aeruginosa* strains, which is consistent with previous research [18]. In our analysis, six of the strains were identified as aligned with the reference strain 34Pae36 in more than 97% of their genomic sequences, suggesting a strong clonal relationship with remarkable genomic conservation. This similarity suggests the presence of clones that maintain robust genetic characteristics, effectively adapting to their environment, as observed in other studies on clonal strains of *P. aeruginosa* [43].

Strain 547256 showed 91.8% alignment with the reference strain ST773, indicating a genetic diversity that could reflect the acquisition of mobile genetic elements, such as phages and genomic islands, contributing to resistance to the six classes of antibiotics studied. This finding is consistent with the theory of “genetic capitalism”, proposed by [44], which postulates that high-risk clones, such as those observed in our study, are more likely to acquire new resistance genes due to antibiotic pressure in hospital settings [2,44]. Additionally, two CRISPR systems were identified in this strain, which highlights the complexity of the adaptive immune system of *P. aeruginosa*. The presence of multiple CRISPR systems indicates the ability of this bacterium to defend itself against invasion by mobile genetic elements, such as phages and plasmids. This genetic feature, which was not observed in other XDR strains in this study, suggests that CRISPR systems could play a key role in defense against hostile environmental situations, an observation that is consistent with previous studies linking the presence of CRISPR in high-risk strains [2].

The identification of *P. aeruginosa* strains with specific genomic characteristics is crucial for the formulation of public health policies that address antibiotic resistance. In this study, it is highlighted that one of the analyzed strains, 547256, presents a high similarity with the ST773 sequence type, which has been associated with multiple resistance genes, including bla VIM-2, bla OXA-10 and, more recently, bla NDM-1. The latter has been reported in the high-risk strain ST773 in several regions, including India and the United States, where its ability to develop resistance to multiple antimicrobials, including tobramycin, ciprofloxacin and levofloxacin, has been demonstrated. At the same time, its susceptibility to colistin has been confirmed [18,45]. The ST773 strain has also shown antibiotic resistance genes (ARGs), including qnrVC1, located in a class I integron, suggesting a potential for horizontal transmission of resistance [46]. A *P. aeruginosa* PA790, belonging to ST773, has been reported from India, this being the 19th such isolate submitted to NCBI and the first complete genome of ST773 from India. WGS data with multiple *P. aeruginosa* (ST773) ARGs will enable understanding of the evolution and phylogeny of these high-risk clones and provide a solid foundation for future research on XDR strains [47]. This finding is important, since strain 547256 represents the first report of ST773 in Colombia, which highlights the need to promote surveillance and control strategies in the context of public health to prevent the dissemination of high-risk clones carrying multiple resistance mechanisms in the country [18].

The identification of resistance genes and virulence factors, such as MexA, MexB and OprM, which make up the MexAB-OprM efflux pump system, together with beta-lactamases, such as AmpC, BlaOXA, BlaVIM and BlaIMP, LasR, RhlR, and PqsR that are regulators of quorum signaling, among others, confirms the ability of these strains to resist a wide range of antibiotics, including carbapenems. These results agree with the findings of [6], who reported that MDR rates were higher in Latin America and Europe, and that, although they have decreased in recent periods, carbapenem resistance remains a significant concern. It is therefore recommended that this type of study be expanded with a greater number of clinical isolates and a broader genomic surveillance approach.

## 5. Conclusions

The *P. aeruginosa* strains analyzed are XDR, with one of them showing high alignment with the reference strain ST773, a high-risk clone not previously reported in Colombia. Additionally, an extensive repertoire of resistance and virulence genes was identified, highlighting its ability to cause difficult-to-treat infections. This genetic profile reinforces the need for more robust and targeted therapeutic approaches for managing infections caused by these strains, as well as the importance of continued surveillance to detect the emergence of new resistance and virulence determinants.

Moreover, the identification of a high-risk clone underscores the potential for the spread of multidrug-resistant lineages within hospital environments, posing a serious challenge for infection control measures. The coexistence of multiple resistance mechanisms within these isolates suggests a limited efficacy of conventional treatments, emphasizing the urgent need for novel therapeutic strategies. Future studies should focus on characterizing the functional impact of these genetic determinants and assessing alternative treatment options to counteract the growing antimicrobial resistance crisis.

## Figures and Tables

**Table 1:** Resistance pattern of the strains evaluated.

Strains	Class	Antibiotic	MIC	Interpretation
544871 572897 629590 635020 637345 645441 547256^1^	Beta-lactam/betalactamase inhibitor	Piperacillin/Tazobactam	≥128	R
		Ceftazidime/Avibactam	≥16	R
	Cephalosporins	Cefazolin	≥64	R
		Ceftazidime	≥64	R
		Cefepime	≥64	R
	Monobactam	Aztreonam	≥64	R
	Carbapenems	Meropenem	≥16	R
	Aminoglycosides	Amikacin	≥64	R
		Gentamicin	≥ 16	R
	Fluoroquinolones	Ciprofloxacin	≥4, 2 ^1^	R, I ^1^
	Glycylcyclines	Tigecycline	≥8	R

^1^ Strain 547256 showed intermediate susceptibility to ciprofloxacin.

**Table 2:** Illumina NovaSeq sequencing results for *P. aeruginosa* isolates.

Strains	Total Number of Sequences	Average Depth	Total Readings	Total Sequence Length (bp)	Q30(%)	GC%	N50 STATISTICS (bp)	Number of CDSs	Integrity Percentage
544871	228	367,544	36,511,708	7,304,619 bp	94.53	65.69%	265,943	6838	99.86%
547256	134	339,786	32,642,984	7,046,366	93.97	65.7%	271,048	6594	99.79%
572897	233	283,907	27,412,470	7,073,876	93.94	66.0%	205,279	6580	99.86%
629590	206	484,218	46,881,312	7,109,622	94.07	65.8%	284,837	6651	99.86%
635020	182	426,345	41,478,374	7,104,378	92.59	65.8%	363,875	6621	99.86%
637345	168	207,998	19,829,196	6,982,541	93.11	65.6%	258,373	6580	97.94%
645441	184	299,912	28,952,368	7,041,958	92.44	65.9%	265,943	6546	99.86%

**Table 3:** Comparative analysis of the different strains of *P. aeruginosa* using BLAST database.

Strains 1	Strains 2	Aligned Bases %	Avg Identity %	Total SNPs
544871	34Pae36 ^1^	97.0824	99.9785	156
547256	ST773 ^2^	91.8498	99.9663	1379
572897	34Pae36	99.2537	99.9828	98
629590	34Pae36	99.5708	99.9859	78
635020	34Pae36	99.1635	99.9788	196
637345	34Pae36	98.7693	99.9829	89
645441	34Pae36	99.1467	99.9747	336

^1^ *P. aeruginosa* strain whose complete genome has been sequenced and deposited in the GenBank database under the accession number CP095770.1. ^2^ High-risk clone of *P. aeruginosa* whose complete genome has been sequenced and deposited in the GenBank database under the accession number NZ_CP041945.1.

**Table 4:** Comparative analysis between different strains of *P. aeruginosa*.

Strains 1	Strains 2	Aligned Bases %	Avg Identity %	Total SNPs
572897	645441	99.0469	99.9871	269
635020	645441	99.0203	99.9918	109
572897	629590	98.9943	99.9945	56
572897	635020	98.9639	99.988	103
629590	635020	98.9449	99.9874	61
629590	645441	98.5915	99.9866	282
637345	645441	97.4426	99.9858	291
544871	635020	96.8277	99.989	125
629590	637345	96.7057	99.9903	44
572897	637345	96.6922	99.987	78
544871	572897	96.6521	99.9896	92
635020	637345	96.6505	99.9863	47
544871	629590	96.4404	99.9907	102
544871	645441	96.2189	99.9872	290
544871	637345	95.0555	99.9897	103
547256	635020	88.6768	98.8711	64,848
547256	572897	88.1132	98.8737	64,629
547256	645441	88.1131	98.8686	64,874
547256	629590	87.6866	98.8643	64,474
547256	637345	86.3051	98.897	61,629
544871	547256	85.685	98.8757	64,831

**Table 5:** Phenotype analysis with respect to the genotype of *P. aeruginosa* strains.

Class	Antibiotic	Related Gene
Beta-lactam with Beta-lactamase inhibitor	Piperacillin/Tazobactam	mexY, mexX, APH(3′)-IIb, OpmH, MexB, mexM, OprM (Resistance: Efflux pump), OXA-395, bcr-1 (Resistance: Beta-lactamases), ArmR (Regulation)
	Ceftazidime/Avibactam	
Cephalosporins	Cefazolin	KPC-2, VIM-2 (Resistance: Beta-lactamases), MexB, OprJ, MexD, opmE, mexN (Resistance: Efflux pump)
	Ceftazidime	
	Cefepime	
Monobactam	Aztreonam	MuxC, MuxA (Virulence: Efflux pump), OpmB, MuxB (Resistance: Efflux pump)
Carbapenems	Meropenem	MexB, opmE, mexN (Resistance: Efflux pump)
Aminoglycosides	Amikacin	mexY, mexX, APH(3′)-IIb, opmE, mexQ, mexN, mexP (Resistance: Efflux pump)
	Gentamicin	
Fluoroquinolones	Ciprofloxacin	MexA, MexE, OprN, MexW, MexV, MexC, MexF, MexI, MexB, MexG, mexM, OprJ, MexH, OprM, MexD, opmE, mexQ, mexN, mexP, MexL (Resistance: Efflux pump), ArmR (Regulation)
Glycylcyclines	Tigecycline	MexX, mexY, OprM (Resistance: Efflux pump)
Quaternary compounds	Benzalkonium chloride	emrE (Resistance: Efflux pump)
Inhibitor of bacterial protein synthesis	Chloramphenicol	catB7, MexI, MexB, OprM, mexQ, mexP, MexL (Resistance: Efflux pump)
Macrolides	Erythromycin	MexB, MexG, MexI, OprJ, MexH, OpmB, MexD, MuxB, OprM, mexQ, mexP, MexL, MexK, MexJ (Resistencia:Bomba de eflujo), MuxC, MuxA (Virulence: Efflux pump), ArmR (Regulation)
Polymyxins	Colistin	arnA (Resistance: Lipid A modification), ParS, basS (Resistance: Two-component systems)
Sulfonamides	Sulfamethoxazole, Sulfadiazine, Sulfisoxazole	sul1 (Resistance: Sulfonamines)
Antiseptic	Triclosan	TriB, TriA, TriC (Resistance: Efflux pump)

## Data Availability

The original contributions presented in this study are included in this article. Further inquiries can be directed to the corresponding authors.

## Abbreviations

The following abbreviations are used in this manuscript:

ANI	Average Nucleotide Identity
WHO	World Health Organization
CR-PA	Carbapenem-resistant *P. aeruginosa*
MDR	Multidrug-resistant
XDR	Extremely drug-resistant
MIC	Minimum inhibitory concentration
CNSG	National Center for Genomic Sequencing
RGI	Resistance Gene Identifier
CARD	Comprehensive Antibiotic Resistance Database
VFDB	Virulence Factor Database
SNPs	Single nucleotide polymorphisms
